# Retrograde intrarenal surgery with intelligent control of renal pelvic pressure for staghorn calculi: a case report

**DOI:** 10.3389/fmed.2024.1321184

**Published:** 2024-01-24

**Authors:** Xiaolong He, Xin Huang, Qiliang Zhai, Leming Song, Xiaolin Deng

**Affiliations:** Department of Urology, The Affiliated Ganzhou Hospital of Nanchang University, Ganzhou, China

**Keywords:** staghorn calculi, intelligent control, retrograde intrarenal surgery, renal pelvic pressure, case report

## Abstract

Percutaneous nephrolithotomy is the gold standard treatment for staghorn calculi. However, this study reviews a case of an almost complete removal of staghorn calculi following one session of retrograde intrarenal surgery with intelligent control of renal pelvic pressure (RIRS-ICP). A 45 years-old female patient with an 8.3 × 4.5 cm complete staghorn stone was infected with *Proteus mirabilis*. Two sensitive antibiotics, piperacillin tazobactam and etimicin, were administered for 3 days. Semirigid 7/8.4 Fr ureteroscope was used to treat the renal pelvis and upper calyceal calculi for 57 min. A 550 μm holmium laser fiber with 2.0 J × 30 Hz was set. Next, a disposable flexible ureteroscope of 8.4 Fr was used to address residual middle and lower calyx stones for 94 min. A 200 μm holmium laser fiber with 1.0 J × 30 Hz was set. The renal pelvis pressure was controlled within 15 mmHg. A 2 mm CT scan on the first postoperative day showed inferior caliceal residue of approximately 1.0 × 0.6 cm. No complications occurred. This suggests that RIRS-ICP is a safe and effective treatment for staghorn calculi.

## Introduction

Staghorn calculi are large, branched kidney stones that occupy a sizable portion of the pyelocaliceal cavities. Most authors agree to differentiate “complete” from “partial” staghorn calculi by the extent of involved calices (1). For staghorn calculi, percutaneous nephrolithotomy (PCNL) is still the first choice for the treatment of kidney stones >2 cm (2). The European Association of Urology guidelines suggest retrograde intrarenal surgery (RIRS)only as a combined approach together with PCNL. With the advent of disposable flexible ureteroscope, RIRS has been selectively used to treat kidney stones larger than 2 cm, especially in patients with solitary kidney. However, the efficiency and safety of conventional RIRS limit its use in staghorn calculi. In particular, elevated renal pelvic pressure (RPP) causes absorption of liquid, bacteria, and endotoxins during surgery, which may lead to fever, sepsis, and shock (3–5).

In order to improve the safety and efficiency of RIRS, we have invented a novel RIRS with intelligent control of renal pelvic pressure (RIRS-ICP) (6, 7). Previous studies have confirmed that the novel technology can significantly improve efficiency and safety (7–11). However, there have been no reports of RIRS-ICP for the treatment of staghorn calculi. In this study, we review a case of an almost complete removal of staghorn calculi following one-stage of RIRS-ICP.

## Case description

**Figure 1:**
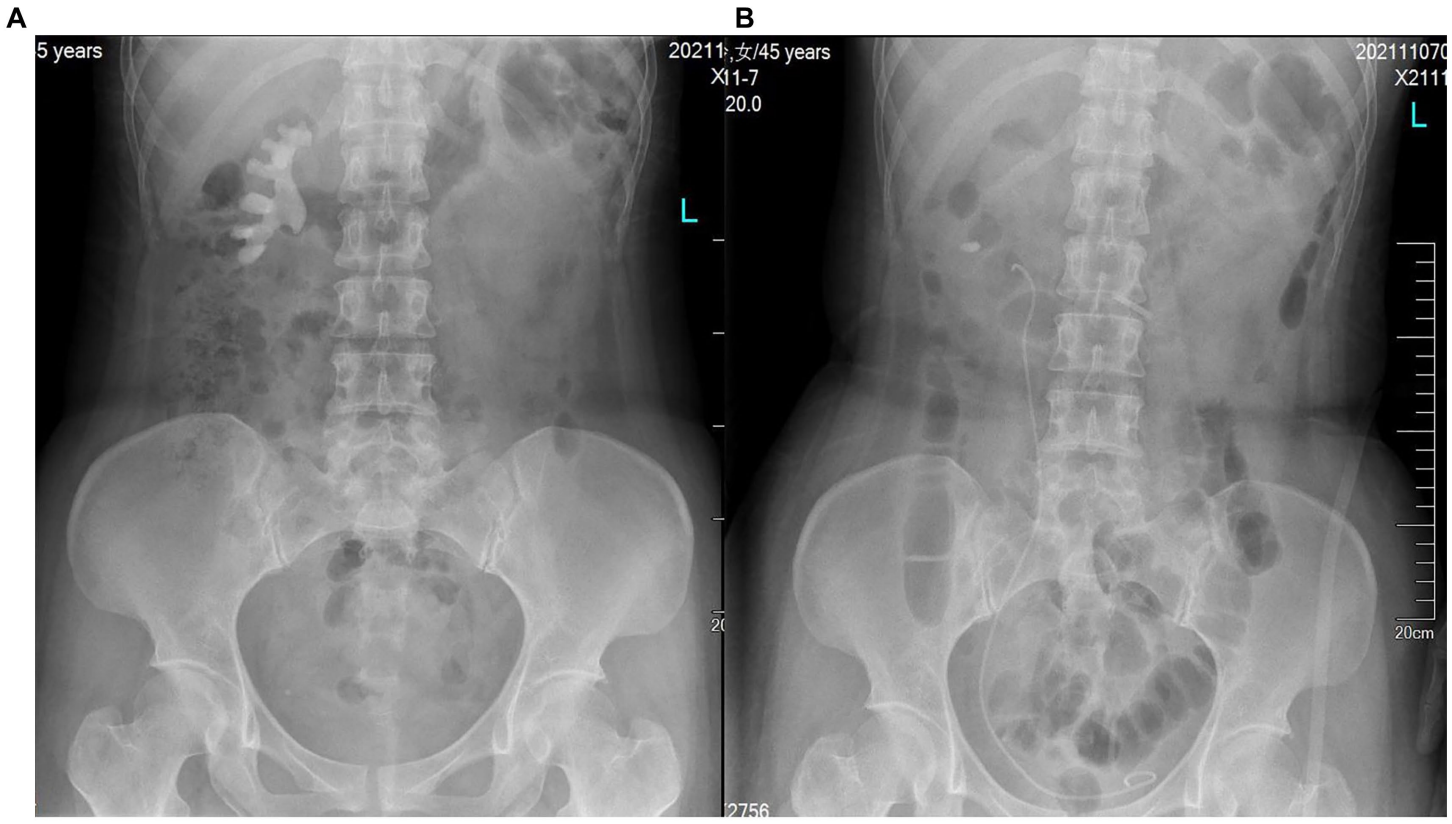
KUB of **(A)** before and **(B)** after surgery.

**Figure 2:**
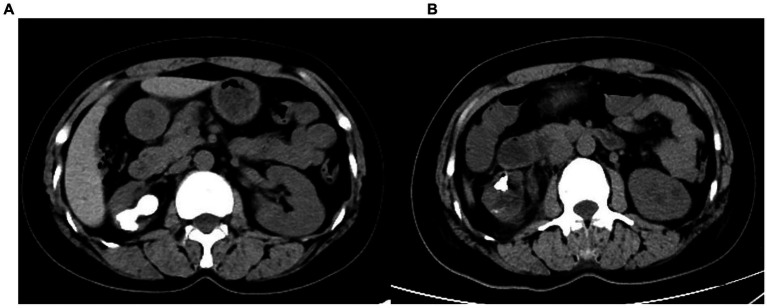
CT of **(A)** before and **(B)** after surgery.

A 45 years-old female patient was admitted to our hospital after 2 years of physical examinations, which diagnosed staghorn calculi in the right kidney. The patient’s physical examination and symptoms were negative. The patient had not received any previous treatment, and was in good health. The double J was not reserved before surgery, and computed tomography (CT) examination suggested an 8.3 × 4.5 cm complete staghorn stone with a CT value of 1,321 Hu (Figures 1A, 2A). A routine urine examination suggested WBC 2405.40/μL, and a bacteria count of 2713.1/μL. Serum creatinine for renal function was normal. A urine bacterial culture suggested *Proteus mirabilis* infection. Two sensitive antibiotics, piperacillin tazobactam and etimicin, were administered for 3 days. Following antibiotics, the WBC count decreased to 696.7/μL in the routine urine examination, while the bacterial count decreased to 73.2/μL.

**Figure 3:**
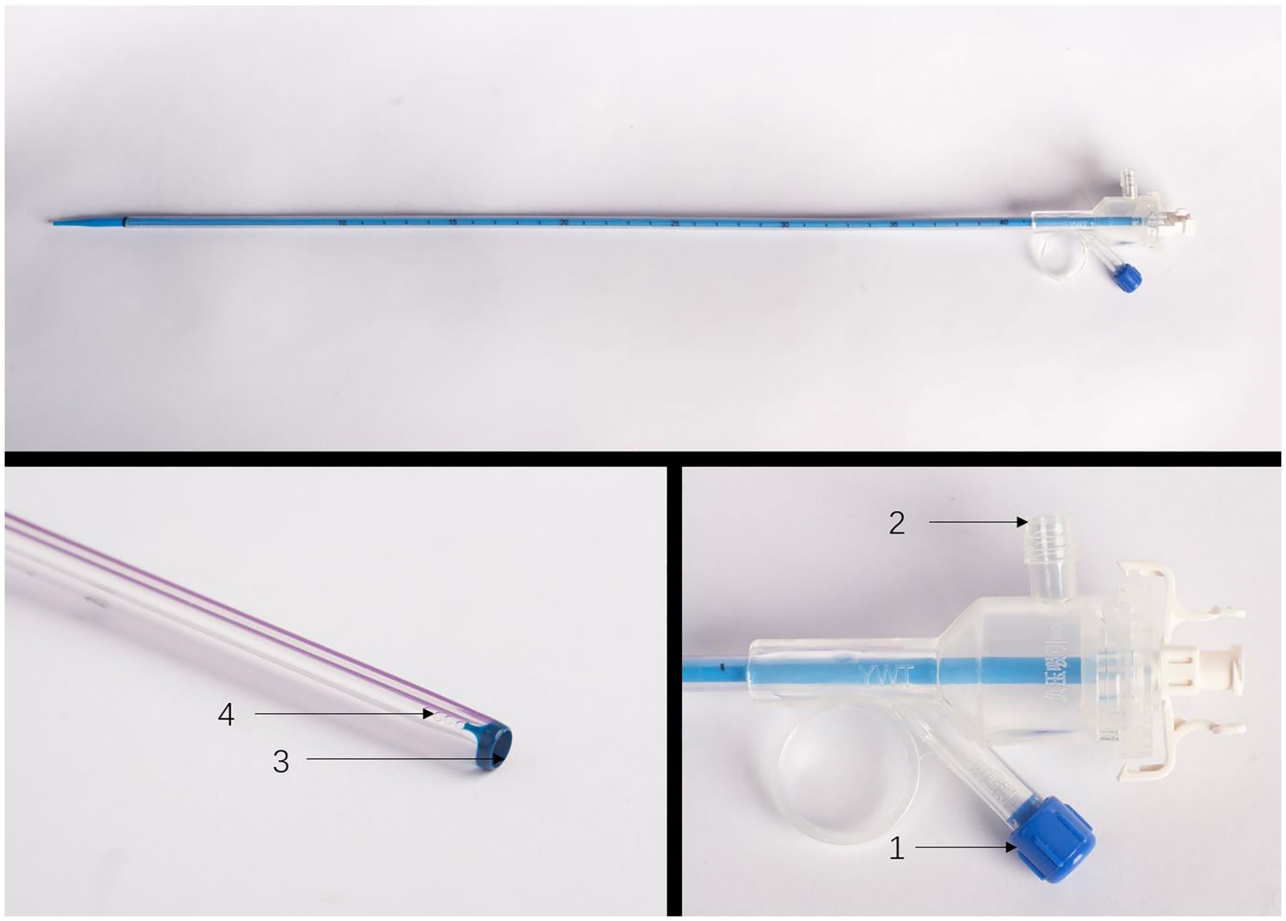
(1) Pressure channel, (2) suctioning channel, (3) working channel, and (4) pressure measuring hole.

**Figure 4:**
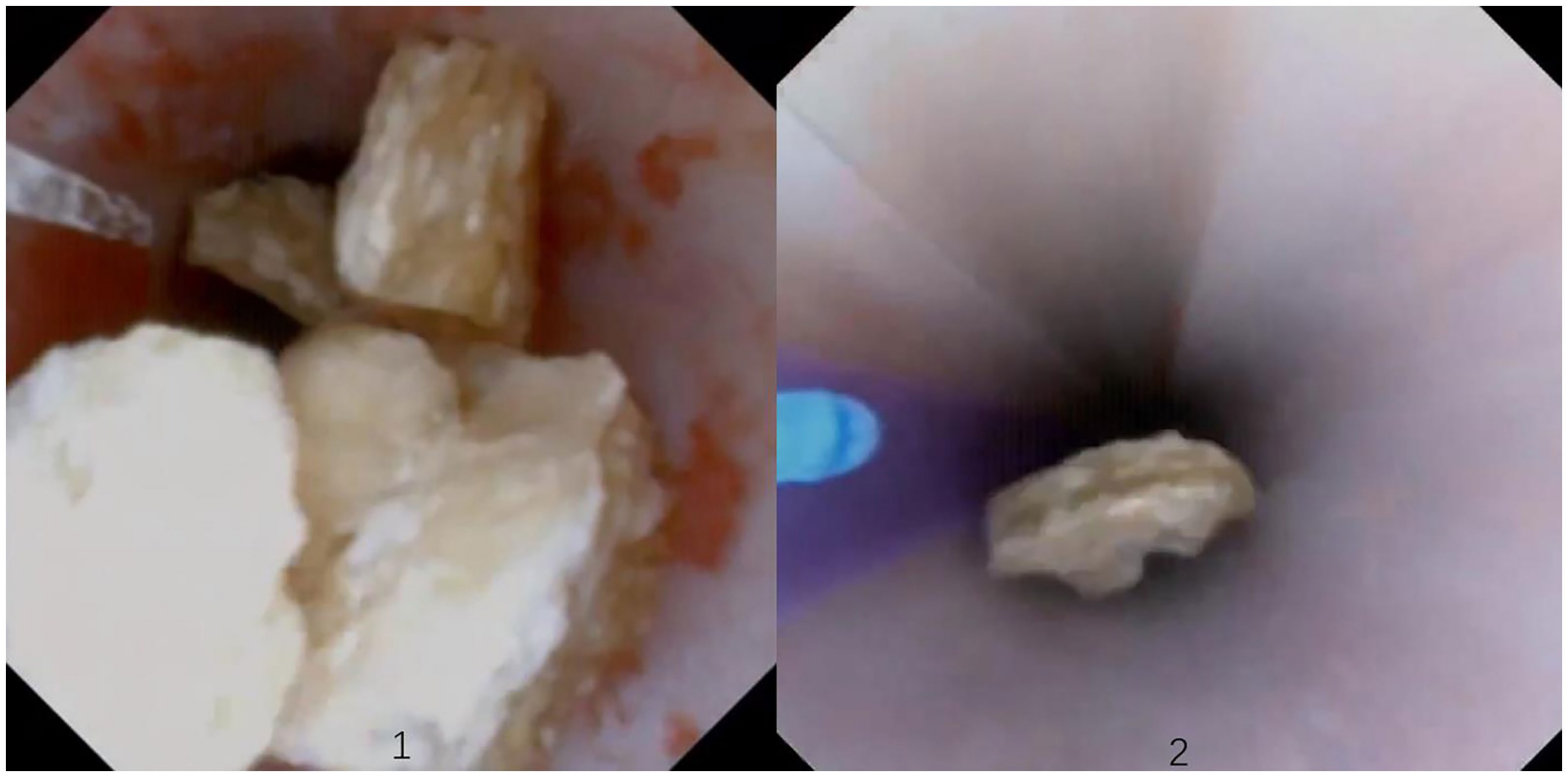
(1) Stones located outside the sheath, (2) stones located in the sheath.

The patient is placed in a lithotomy-oblique supine position with the suffering kidney on top and 60° on the lateral side. Initially, a semirigid 7/8.4 Fr ureteroscope (KARL Storz, Germany) was used for ureteroscopy. There is an irrigation channel of 2.4 Fr and a working channel of 3.4 Fr of the rigid ureteroscope. The main part of the renal pelvic calculus is within the visual field of the rigid ureteroscope. Therefore, the patented ureteral access sheath (UAS, 12/14 Fr, 35 cm length, Figure 3) with pressure measurement and suction was placed along the guidewire. The platform selection mode was set to fully automatic. The pressure and suctioning channels of the UAS were connected to the irrigation and suctioning platform via a pressure sensing pipe and suctioning pipe. After the sensing pipe is injected with normal saline using a syringe, the normal saline and urine in the renal pelvis are drained through the sheath to completely empty the air in the pressure sensor pipe for accurate pressure measurement. After that, zero calibration was performed at the platform. The actual pressure in the renal pelvis displayed on the platform was 0 mmHg. The perfusion flow was initially set at 100 mL/min and adjusted according to intraoperative conditions. The RPP control value was set to −15 to −5 mmHg. The RPP alarm value was set to 15 mmHg. A 550 μm Holmium laser fiber (Lumenis) was used with the power 2.0 J × 30 Hz. Ureteroscopy was used to treat the renal pelvis and upper calyceal calculi within the visual field for 57 min. Following this, a disposable flexible ureteroscope of 8.4 Fr was used to deal with residual middle and lower calyx stones for 94 min. A 200 μm Holmium laser fiber (Lumenis) was used with the power was set to 1.0 J × 30 Hz. When blood clots or stone powder blocked the outlet, the actual renal pelvis pressure exceeded 10 mmHg, and the platform sounded an alarm. Gravel particles larger than sheath gap but less than sheath in diameter were sucked out by withdrawing the scope intermittently without a need of stone basketing (Figure 4). The platform directly stopped the perfusion when the actual renal pelvis pressure exceeded 15 mmHg. No ureteral perforation, avulsion or other complications occurred during the operation. The routine blood panel was normal immediately after the operation. The C-reactive protein level was 8.00 mg/L, and procalcitonin was <0.01 ng/mL after surgery. The total number of WBCs was 13.89*109, C-reactive protein was 54.41 mg/L and procalcitonin was 0.53 ng/mL on the first day after surgery. A 2 mm CT scan and KUB on the first postoperative day showed inferior caliceal residue of approximately 1.0 × 0.6 cm and no perirenal exudation or bleeding (Figures 1B, 2B). Stone composition analysis suggested carbonate apatite and calcium oxalate monohydrate. The ureteral stent was removed 1 month after the operation, and no ureteral stricture occurred 3 months post-operation by ultrasound showing no hydronephrosis. Because the patient did not consent to a second surgical time or an eswl treatment. Ultrasound and CT were checked regularly.

The study was approved by Ethics Committee of the Ganzhou People’s Hospital. The study was conducted in accordance with the local legislation and institutional requirements. The participants provided their written informed consent to participate in this study.

## Discussion

Urolithiasis is a common urological disease, affecting 10%–15% of the population (12). Approximately 10%–20% of urinary calculi are staghorn calculi. Thirty-six percent of staghorn stones developed significant renal damage after 10 years of conservative treatment, and 49%–68% of these are infectious. The incidence of staghorn calculi is higher in women than that in men (13). It consists of struvite and is associated with recurrent urinary tract infection (14). Conservative treatment of staghorn calculi often leads to renal impairment and sepsis, with a mortality rate of 30% (15). Therefore, staghorn calculi should be actively treated with surgery. The goal of surgical treatment for staghorn calculi is the complete removal of the stones to prevent recurrence and to maximize the protection of renal function. Due to its high stone clearance rate, PCNL has been recommended as the most suitable method for staghorn calculi. Despite the high stone-free rate of PCNL, there are shortcomings, such as channel damage to renal parenchyma, renal hemorrhage and infection, long hospital stays, and slow recovery. PCNL is riskier, especially in special patients, including those with no hydronephrosis kidney, combined with cardiovascular and cerebrovascular complications, solitary kidney, and patients with coagulation dysfunction (16, 17). Multichannel PCNL was thought to come with more complications, which could be particularly important factors for solitary kidney patients (18).

In recent years, ureteroscopy has often been used to treat upper urinary calculi because it passes through the natural orifices of the human body, and a flexible ureteroscope can reach each calyx, with the advantages of less surgical trauma and quick recovery. RIRS is recommended for the treatment of kidney stones smaller than 2.0 cm. With the advent of disposable flexible ureteroscope, RIRS has been attempted in special staghorn stones, including residual fragments after PCNL, requiring anti-coagulation, urinary tract deformation, and solitary kidney (19–21). Following the first operation attempt, stone clearing rates may be low, but an increase in stone clearing rates has been documented following multiple operations; however, the risk of complications associated with multiple procedures, such as infection and the economic burden on patients, increases with each operation (22). The reasons for this may include the low efficiency, the high renal pelvic pressure, which is prone to infection, fluid absorption and other complications (5). Low perfusion flow also resulted in ureteral stricture due to holmium laser thermal injury (23).

How can we break up and remove stone gravel safely and efficiently under a high flow rate? First, high-power holmium laser equipment is necessary. Second, we have developed a novel RIRS-ICP technique. The platform measures RPP through the sheath 6 times per second. The platform automatically controls attraction through pressure feedback so that the RPP always fluctuates up and down the set value at different flow rates. When the RPP exceeds the warning value for various reasons, the platform will sound an alarm and automatically stop perfusion (24). This technology solves the problem reported by Doizi et al. (25) that pressure can only be measured but not controlled. Previous studies have confirmed that this technology can significantly improve efficiency and safety (5, 11).

In this case, the patient had a complex medical presentation manifesting as a staghorn calculus 8.3 × 4.5 cm in size with uncontrolled infection. A preoperative CT showed that the patient’s renal calculus filled the renal pelvis and each calyx, forming a complete staghorn calculus. Each calyx stone was close to the renal surface, and each calyx was not connected to each other. Renal atrophy was observed. If the standard channel method is adopted, calculi cannot be removed in one stage, and postoperative surgery or external lithotripsy is needed. Multiple channels of PCNL aggravate the risk of renal function damage and bleeding. A single application of traditional flexible ureteroscopy requires multiple stages, and each stage requires a long operation time, resulting in a high risk of infection and high cost. Patients with positive urine culture bacteria need to be negative before surgery in accordance with the guidelines. This is based on the factor that even with continued use of anti-infective therapy, negative urine culture bacteria does not mean that the kidney stone is free of bacteria or that the small calyces of stone obstruction are free of bacterial infection. To prevent further renal damage caused by bacterial infection, we only administered 3 days of anti-infection treatment before surgery. CT and KUB showed that the renal pelvis, middle and upper calyces, and ureter were at the same level. Rigid ureteroscopic lithotripsis can be used first for 2/3 of the total stone load.

The patient requested RIRS as much as possible, indicating that the second or third stage of surgery could be accepted. After a full discussion with the department team, RIRS-ICP was selected to address the case. First, rigid and flexible ureteroscopes were used in combination. In the first stage, a rigid ureteroscope was used, and 150 mL high flow perfusion was used under safe pressure. The operation proved convenient, also a 550 μm high-power holmium laser can be used, similar to a long-channel PCNL operation, which greatly improves the efficiency of gravel and lithotomy. When a rigid ureteroscope could not see the calculi, a disposable flexible ureteroscope was used with 200 μm fiber. Although the lithotripy efficiency was lower, flexible ureteroscopy could check the calculi of each calyceum and reduce the residual calculi to the greatest extent. Second, the lithotomy-oblique supine position was used instead of the lithotomy position. In the oblique supine lithotomy position, the outlet of the renal pelvis is the lowest position of the collecting system. The stone particles can easily fall into the renal pelvis and sheath under the effect of gravity and high-flow perfusion, which significantly improves the lithotripsy efficiency (10). Kidney stones were removed in almost one stage from the natural orifice. There were no complications, such as fluid extravasation, fever and sepsis, during the long postoperative period, which was most likely due to the effective control of intraoperative renal pelvis pressure.

Finally, the reason for reviewing a female patient case in this study was to explore the use of the rigid ureteroscope, which is 43 cm in length and can only be inserted into the 35 cm sheath to reach the renal pelvis and upper calyces. It is known to be suitable for upper ureteral calculi in male patients and was thus tested in females. For kidney calculi, a customized and extended rigid ureteroscope of 10 cm must be used. In addition, the key to successful operation is the placement of the sheath into the renal pelvis or upper calyces, which is conducive to crushing the stone if immediately vacuumed out. Therefore, preoperative imaging and ureteroscopy are very important to evaluating the angle and motion of the upper calyces, renal pelvis and ureter. For patients with difficulty in placing the sheath into the pelvis, the assistant should extrude the abdomen to expand the lower pole, thus reducing the difficulty of placing the sheath into the pelvis. For blocked stones, the assistant can pat the renal area to promote the loosening, tossing, and migration of stone particles to the renal pelvis. If the sheath is difficult to place into the pelvis, a flexible ureteroscope should be performed immediately, but the operation time may be significantly longer.

It should be pointed out that this study is only a case report, and the safety and effectiveness of this technique compared with percutaneous nephrolithotomy is not strictly designed in the control group, which requires further study.

## Data availability statement

The original contributions presented in the study are included in the article/supplementary material, further inquiries can be directed to the corresponding author.

## Ethics statement

The studies involving humans were approved by Ethics Committee of the Ganzhou People’s Hospital. The studies were conducted in accordance with the local legislation and institutional requirements. The participants provided their written informed consent to participate in this study. Written informed consent was obtained from the individual(s) for the publication of any potentially identifiable images or data included in this article.

## Author contributions

XHe: Writing – original draft. XHu: Conceptualization, Investigation, Writing – review & editing. QZ: Data curation, Writing – original draft. LS: Conceptualization, Investigation, Writing – review & editing. XD: Supervision, Writing – review & editing, Investigation.

## References

[1] Keller EX De Coninck V Doizi S Traxer O. The role of ureteroscopy for treatment of staghorn calculi: a systematic review. Asian J Urol. (2020) 7:110–5. doi: 10.1016/j.ajur.2019.10.012, PMID: 32257803 PMC7096690

[2] Türk C Petřík A Sarica K Seitz C Skolarikos A Straub M. EAU guidelines on interventional treatment for urolithiasis. Eur Urol. (2016) 69:475–82. doi: 10.1016/j.eururo.2015.07.041 26344917

[3] Tokas T Herrmann TRW Skolarikos A Nagele U Training and Research in Urological Surgery and Technology (T.R.U.S.T.)-Group. Pressure matters: intrarenal pressures during normal and pathological conditions, and impact of increased values to renal physiology. World J Urol. (2019) 37:125–31. doi: 10.1007/s00345-018-2378-4, PMID: 29915945

[4] Tokas T Skolarikos A Herrmann TRW Nagele U Training and Research in Urological Surgery and Technology (T.R.U.S.T.)-Group. Pressure matters 2: intrarenal pressure ranges during upper-tract endourological procedures. World J Urol. (2019) 37:133–42. doi: 10.1007/s00345-018-2379-3, PMID: 29915944

[5] Tokas T Tzanaki E Nagele U Somani BK. Role of intrarenal pressure in modern day endourology (mini-PCNL and flexible URS): a systematic review of literature. Curr Urol Rep. (2021) 22:52–5. doi: 10.1007/s11934-021-01067-5, PMID: 34622341

[6] Deng X Song L Xie D Zhu L Yao L Huang J. Suctioning flexible ureteroscopy with automatic control of renal pelvic pressure. J Urol. (2015) 29:e782–2. doi: 10.1089/vid.2015.0036 35220314

[7] Zhu X Song L Xie D Peng Z Guo S Deng X. Animal experimental study to test application of intelligent pressure control device in monitoring and control of renal pelvic pressure during flexible ureteroscopy. Urology. (2016) 91:242.e11–5. doi: 10.1016/j.urology.2016.02.022 26919967

[8] Deng X Song L Xie D Fan D Zhu L Yao L. A novel flexible ureteroscopy with intelligent control of renal pelvic pressure: an initial experience of 93 cases. J Endourol. (2016) 30:1067–72. doi: 10.1089/end.2015.0770 27558001

[9] Chen H Qiu X Du C Xie D Liu T Wang G. The comparison study of flexible ureteroscopic suctioning lithotripsy with intelligent pressure control versus minimally invasive percutaneous suctioning nephrolithotomy in treating renal calculi of 2 to 3 cm in size. Semin Laparosc Surg. (2019) 26:528–35. doi: 10.1177/1553350619849782, PMID: 31130072

[10] Peng G Song L Xie D Huang J Zhong Y Tan W. Suctioning flexible ureteroscopic lithotripsy in the oblique supine lithotomy position and supine lithotomy position: a comparative retrospective study. Minerva Urol Nefrol. (2018) 70:612–6. doi: 10.23736/S0393-2249.18.03144-2, PMID: 30037207

[11] Huang J Xie D Xiong R Deng X Huang C Fan D. The application of suctioning flexible ureteroscopy with intelligent pressure control in treating upper urinary tract calculi on patients with a solitary kidney. Urology. (2018) 111:44–7. doi: 10.1016/j.urology.2017.07.042, PMID: 28802568

[12] Desai M Sun Y Buchholz N Fuller A Matsuda T Matlaga B. Treatment selection for urolithiasis: percutaneous nephrolithomy, ureteroscopy, shock wave lithotripsy, and active monitoring. World J Urol. (2017) 35:1395–9. doi: 10.1007/s00345-017-2030-8, PMID: 28303335

[13] Johnson CM Wilson DM O'Fallon WM Malek RS Kurland LT. Renal stone epidemiology: a 25-year study in Rochester, Minnesota. Kidney Int. (1979) 16:624–31. doi: 10.1038/ki.1979.173, PMID: 548606

[14] Alsawi M Amer T Mariappan M Nalagatla S Ramsay A Aboumarzouk O. Conservative management of staghorn stones. Ann R Coll Surg Engl. (2020) 102:243–7. doi: 10.1308/rcsann.2019.0176, PMID: 31918554 PMC7099166

[15] Teichman J Long R Hulbert J. Long-term renal fate and prognosis after staghorn calculus management. J Urol. (1995) 153:1403–7. doi: 10.1016/S0022-5347(01)67413-5, PMID: 7714951

[16] Gadzhiev N Malkhasyan V Akopyan G Petrov S Jefferson F Okhunov Z. Percutaneous nephrolithotomy for staghorn calculi: troubleshooting and managing complications. Asian J Urol. (2020) 7:139–48. doi: 10.1016/j.ajur.2019.10.004, PMID: 32257807 PMC7096695

[17] Shi X Peng Y Li L Li X Wang Q Zhang W. Renal function changes after percutaneous nephrolithotomy in patients with renal calculi with a solitary kidney compared to bilateral kidneys. BJU Int. (2018) 122:633–8. doi: 10.1111/bju.14413, PMID: 29802813

[18] Liu C Cui Z Zeng G Wan SP Li J Zhu W. The optimal minimally invasive percutaneous nephrolithotomy strategy for the treatment of staghorn stones in a solitary kidney. Urolithiasis. (2016) 44:149–54. doi: 10.1007/s00240-015-0803-3, PMID: 26209008

[19] Shi X Peng Y Li X Wang Q Li L Liu M. Propensity score-matched analysis comparing retrograde intrarenal surgery with percutaneous nephrolithotomy for large stones in patients with a solitary kidney. J Endourol. (2018) 32:198–204. doi: 10.1089/end.2017.0482, PMID: 29212373

[20] Sfoungaristos S Mykoniatis I Katafigiotis I Isid A Gofrit ON Constantinides CA. Single lower calyceal percutaneous tract combined with flexible nephroscopy: a valuable treatment paradigm for staghorn stones. Can Urol Assoc J. (2018) 12:E21–4. doi: 10.5489/cuaj.4393, PMID: 29173274 PMC5783703

[21] Jiang K Zhang P Xu B Luo G Hu J Zhu J. Percutaneous nephrolithotomy vs. retrograde intrarenal surgery for renal stones larger than 2 cm in patients with a solitary kidney: a systematic review and a meta-analysis. Urol J. (2020) 17:442–8. doi: 10.22037/uj.v16i7.5609, PMID: 32748387

[22] Birowo P Rasyid N Atmoko W Sutojo B. Case report: An occurrence of steinstrasse in retrograde intra renal surgery (RIRS) for large staghorn kidney stone: a difficult experience in managing surgical outcomes. F1000Res. (2020) 9:184–4. doi: 10.12688/f1000research.22448.2 32724559 PMC7338919

[23] Winship B Wollin D Carlos E Peters C Li J Terry R. The rise and fall of high temperatures during ureteroscopic holmium laser lithotripsy. J Endourol. (2019) 33:794–9. doi: 10.1089/end.2019.0084, PMID: 31016991

[24] Deng X Song L Xie D Huang J Zhu L Su G. Suctioning flexible ureteroscopy with automatic control of renal pelvic pressure: a porcine model. Int J Clin Exp Med. (2016) 9:6563–8.

[25] Doizi S Letendre J Cloutier J Ploumidis A Traxer O. Continuous monitoring of intrapelvic pressure during flexible ureteroscopy using a sensor wire: a pilot study. World J Urol. (2021) 39:555–61. doi: 10.1007/s00345-020-03216-w, PMID: 32382840

